# Taxonomic position of *Eriocycla* (Apiaceae): insights from molecular and morphological evidence

**DOI:** 10.1093/aobpla/plaf045

**Published:** 2025-08-28

**Authors:** Yang-Zhao Li, Jing Cai, Zi-Xuan Li, Xing-Jin He, Song-Dong Zhou

**Affiliations:** Key Laboratory of Bio-Resource and Eco-Environment of Ministry of Education, College of Life Sciences, Sichuan University, Chengdu 610065, Sichuan, China; Key Laboratory of Bio-Resource and Eco-Environment of Ministry of Education, College of Life Sciences, Sichuan University, Chengdu 610065, Sichuan, China; Key Laboratory of Bio-Resource and Eco-Environment of Ministry of Education, College of Life Sciences, Sichuan University, Chengdu 610065, Sichuan, China; Key Laboratory of Bio-Resource and Eco-Environment of Ministry of Education, College of Life Sciences, Sichuan University, Chengdu 610065, Sichuan, China; Key Laboratory of Bio-Resource and Eco-Environment of Ministry of Education, College of Life Sciences, Sichuan University, Chengdu 610065, Sichuan, China; Evolution & Diversity

**Keywords:** Apiaceae, *Seseli*, *Eriocycla*, morphology, phylogeny

## Abstract

Speciation arises from multifaceted factors, making phenotype-based classifications unreliable. Integrative taxonomy has advanced significant breakthroughs in taxonomically challenging groups like Apiaceae, which is characterized by highly convergent morphological traits across species. The genus *Eriocycla* (Apiaceae) has long presented persistent taxonomic uncertainties. While morphological similarities initially supported *Eriocycla* as *Seseli* sect. *Eriocycla*, phylogenetic studies consistently resolve *Eriocycla* within the tribe Echinophoreae, contrasting with *Seseli* (tribe Selineae). Integrated morphological and molecular analyses were conducted here to resolve this taxonomic conflict. Phylogenetic reconstructions based on nuclear ribosomal DNA and plastomes all supported that *Seseli delavayi* and *Seseli nortonii* formed a stable monophyletic group with *Eriocycla nuda* and *Eriocycla pelliotii* within Echinophoreae, separate from *Seseli*. Plastome comparisons across 14 taxa revealed structural conservation in *E. nuda*, *E. pelliotii*, *S. delavayi*, and *S. nortonii*, particularly in inverted-repeat and single-copy regions, distinct from that of other *Seseli* species. A unique inversion involving the *trnY–GUA*, *trnD–GUC*, and *trnE–UUC* genes was detected in *E. nuda* and *E. pelliotii* but absent in *S. delavayi* and *S. nortonii*. Shared morphological characteristics, including glabrous stem bases, basally free bracteoles, and prominent calyx teeth, further support their affinity with *Eriocycla*. We therefore propose to recognize *Eriocycla* as a separate genus rather than as *Seseli* sect. *Eriocycla* and reclassifying *S. delavayi* and *S. nortonii* into it. In conclusion, this study not only revealed the phylogenetic position of the tribe Echinophoreae but also resolved the long-standing taxonomic controversy surrounding *Eriocycla* and *Seseli*.

## Teaser

This study addresses the long-standing taxonomic uncertainties surrounding the genus Eriocycla (Apiaceae), which has often been classified within Seseli due to morphological similarities. Using both morphological and molecular data, including nuclear ribosomal DNA and plastomes, we resolve Eriocycla as a distinct genus within the tribe Echinophoreae. Phylogenetic analyses confirm that Eriocycla species, along with Seseli delavayi and Seseli nortonii, form a monophyletic group separate from Seseli. Plastome comparisons and shared morphological traits further support this reclassification. This work clarifies the evolutionary relationships within Echinophoreae and resolves the taxonomic issues surrounding Eriocycla.

## Introduction

The plants of Apiaceae family, widely distributed over the world, are characterized by (compound) umbels and mericarps (Pimenov and Leonov 1993). Due to convergent evolution and hybridization (Dávalos et al. 2014, Zou and Zhang 2016), related genera often exhibit highly similar morphological features. Consequently, traditional classification systems based solely on phenotypic traits frequently fail to reflect the true phylogenetic relationships within the family (Drude 1898). With the advent of molecular biology, an increasing number of molecular markers, including the internal transcribed spacer (ITS), external transcribed spacer (ETS), and *matK* gene, have been employed to investigate phylogenetic relationships in Apiaceae (Spalik et al. 2004, Valiejo-Roman et al. 2006, Zhou et al. 2009, Downie et al. 2010, Doğan Güner and Duman 2013, Xu et al. 2021, Song et al. 2025). Numerous groups have been demonstrated to be polyphyletic, including the genera *Peucedanum* L., *Ligusticum* L., and *Angelica* L. (Zhou et al. 2020, Jiang et al. 2022, Liu et al. 2024a, 2024b, Ren et al. 2025). These genera share characteristics such as broad geographical distributions, high species diversity, and indistinct morphological boundaries with related groups. While phylogenetic methods can reveal relationships among species, they often fail to provide stable diagnostic characters. Therefore, the integrative taxonomy (synthesizing morphological, phylogenetic, genomic, and complementary methods) has emerged as a major trend in recent taxonomic studies (Dewar et al. 2025, He et al. 2025, Yuan et al. 2025). Integrative taxonomy not only reconstructs evolutionary relationships from multifaceted evidence but also establishes stable morphological boundaries, providing a foundation for field identification.

As one of the largest genera in Apiaceae, *Seseli* comprises 125–140 species and is distributed in the Old World from western Europe and northwestern Africa to China and Japan (Pimenov and Leonov 1993, 2004, Plunkett et al. 2018). The genus was characterized by: absence or abundance of linear, acuminate bracts exceeding pedicels in length, calyx teeth absent, and ovate striate fruits (Linné and Salvius 1754). Due to its wide distribution across diverse climate zones and diverse habitats, this genus exhibits remarkable morphological diversity, and its taxonomy has been debated since its establishment (Drude 1898, Schischkin 1950, Pimenov et al. 2012, Pimenov 2017, Liu et al. 2024a, 2024b). A central controversy involves whether the genus *Eriocycla* should be merged into *Seseli*.

*Eriocycla* Lindl., established with *Eriocycla nuda* Lindl. as the type species, was initially characterized by Lindley (1835) based on its densely pubescent fruits, prominent ribs, solitary vittae in each furrow, and two vittae on the commissure. However, overlapping fruit characteristics with *Pituranthos* Viv., particularly in mericarp morphology, led Clarke (1879) to synonymize *Eriocycla* under *Pituranthos*. Wolff (1927) reinstated and expanded *Eriocycla*, transferring six species into this genus. Comparative fruit anatomical studies of nine *Eriocycla* species revealed that *Eriocycla* and *Seseli* members do not differ in carpological features up to the genus level (Pimenov and Kljuykov 2000). Consequently, *Eriocycla* was reclassified as *Seseli* sect. *Eriocycla* (Lindl.) Pimenov & Kljuykov, with *E. nuda* designated as the section’s type species, and *Seseli nortonii* Fedde ex H.Wolff was treated as a synonym of *E. nuda* due to morphological similarities. According to extensive morphological examination of specimens, Pimenov (2017) synonymized all Chinese *Eriocycla* members with *Seseli*.

Early phylogenetic analyses using nrITS, *rpl16*, and *rps16* intron regions resolved most *Seseli* species within the tribe Selineae (Zhou et al. 2009, Banasiak et al. 2013). However, Lyskov et al. (2022) employed ITS + ETS and *psbD*–t*rnT* sequences to reconstruct the phylogeny of *Seseli*, placing *E. nuda* within the tribe Echinophoreae. Based on ITS sequences, Degtjareva et al. (2011) inferred the phylogenetic positions of 36 *Seseli* species, revealing that *Seseli delavayi* Franch., a narrow endemic to the Hengduan Mountains, was resolved within Echinophoreae.

According to field observations and the checking of collected materials in the type locality, we observed that *S. nortonii* and *S. delavayi* showed shared characteristics with *E. nuda* and *Eriocycla pelliotii*, including glabrous stem bases, basally free bracteoles, and prominent calyx teeth—features distinct from *Seseli tortuosum* and other *Seseli* taxa (Doğan Güner and Duman 2013). Recent studies have demonstrated that pollen characteristics serve as crucial evidence for resolving taxonomic problems in Apiaceae (Xiao et al. 2021, Song et al. 2024, 2025). However, comprehensive morphological investigations for *Eriocycla* are scarce, with only preliminary observations reported by Shu and Sheh (2001). Morphological and molecular similarities indicate the need for additional evidence to re-evaluate the taxonomic position of *Eriocycla* and related *Seseli* taxa.

Drawing on recent revisions in Apiaceae (Cai et al. 2022, Liu et al. 2024a, 2024b, Ren et al. 2025, Song et al. 2025), 3 concatenated datasets were obtained for phylogenetic reconstructions: 34 nuclear ribosomal DNA (nrDNA) (ITS + ETS), 35 plastid fragments (*matK* + *rbcL* + *rps16* intron), and 42 plastomes [79 shared coding sequences (CDS)]. Comparative analyses of plastomes from 14 *Eriocycla* and *Seseli* species were conducted, supplemented by morphological and anatomical characteristics. With the integrated evidence, we aimed to (i) re-evaluate the taxonomic status of *Eriocycla*; (ii) reveal plastid characteristics of *Eriocycla* and related *Seseli* taxa; and (iii) provide new insights into the phylogeny of *Eriocycla* and *Seseli*, resolve the long-standing controversy between these genera.

## Materials and methods

### Taxon materials

Field surveys were conducted near the type localities of four species: *E. nuda* (Purang County, Xizang, China), *E. pelliotii* (Wushi County, Xinjiang, China), *Seseli d.* (Yuanmou County, Yunnan, China), and *Seseli n.* (Tingri County, Xizang, China), with over 10 individuals per species collected for morphological and molecular analyses.

Freshly collected leaves were dried in silica gel and stored. Voucher specimens referenced in this study are deposited at the Herbarium of Sichuan University (S.-D.Z.) (see Supplementary Table S1).

### Morphological observation

Habitat photographs and external morphological features of *E. nuda*, *S. nortonii*, and *S. delavayi* were documented during field surveys. Microstructural observations of inflorescences (bracts, calyx teeth, petals) and mature mericarps (shape, ribs, vittae) were conducted using a stereomicroscope (SMZ25, Nikon Corp., Tokyo, Japan). For pollen morphology, 10 dry, mature, fully developed anthers per species were selected to examine gross morphology (equatorial view, polar view), germinal furrow structure, and exine ornamentation under a JSM-7500F scanning electron microscope. Pollen dimensions were measured using MATO v2.1 (Liu et al. 2023) with 10 replicates per species.

Morphological comparisons of *S. nortonii* and *S. delavayi* with other *Seseli* and *Eriocycla* taxa were based on specimen information, Flora of China (2005), and relevant literature (Linné and Salvius 1754, Pimenov and Kljuykov 2000, Doğan Güner et al. 2011, Doğan Güner and Duman 2013, Cai et al. 2022). Morphological terminology was followed according to Kljuykov et al. (2004), Ostroumova (2021), and Shu and Sheh (2001) .

### DNA extraction, sequencing, assembly, and annotation

From our accumulated materials, 2 *Eriocycla* species (including the type species *E. nuda* and *E. pelliotii*) and 13 *Seseli* taxa (*S. nortonii*, *S. delavayi*, *Seseli mairei*, *Seseli intramongolicum*, *Seseli aemulans*, *Seseli asperulum*, *Seseli coronatum*, *Seseli eriocephalum*, *Seseli glabratum*, *Seseli incisodentatum*, *Seseli squarrulosum*, *Seseli valentinae*, and *Seseli yunnanense*) were selected, representing >80% coverage of both genera’s diversity in China. Total genomic DNA of these 15 species was extracted from silica gel-dried leaves using the modified CTAB method (Pahlich and Gerlitz 1980). Then, we used the primers ITS-4 (5′-TCCTCCGCTTATTGATATGC-3′), ITS-5 (5′-GGAAGTAAAAGTCGTAACAAGG-3′) (White et al. 1990), 18S-ETS (5′-ACTTACACATGCATGGCTTAATCT-3′) (Baldwin and Markos 1998), and Umb-ETS (5′-GCGCATGAGTGGTGAWTKGTA-3′) (Logacheva et al. 2010) to amplify the ITS and ETS regions. Polymerase chain reactions (PCRs) were conducted in 30 μl volume reactions with 2 μl plant total DNA, 1.5 μl forward primer, 1.5 μl reverse primer, 15 μl volume 2× Taq MasterMix (CWBIO, Beijing, China), and 10μl ddH_2_O. The PCR amplification programme was set as follows: initial denaturation at 94°C for 4 min, followed by 36 cycles (denaturation at 94°C for 45 s, annealing at 52°C for 70 s, and extension at 72°C for 90 s), and a final extension at 72°C for 10 min. All PCR products were sent to Sangon (Shanghai, China) for sequencing. Then, the software DNASTAR SeqMan Pro v7.1.0 (Burland 2000) were used to assemble the ITS and ETS sequences.

For plastomes, total genomic DNA libraries of 15 species were generated via Illumina platform, and the 150 bp paired-end reads were created at Novogene (Beijing, China). We employed fastP v0.15.0 (-n 10 and -q 15) to filter the raw data (Chen et al. 2018), and high-quality reads were assembled for the whole plastomes by GetOrganelle v1.7.7.0 (Jin et al. 2020). Genome annotation was performed using Plastid Genome Annotator (Qu et al. 2019) with *Seseli montanum* (KM035851) as the reference. Then, we conducted manual refinements and extracted the *matK* gene, *rbcL* gene, and *rps16* intron from annotated plastomes using Geneious Prime v9.0.2 (Kearse et al. 2012). Plastome circular maps for *E. nuda*, *E. pelliotii*, *S. delavayi*, and *S. nortonii* were generated via the online programme CHLOROPLOT (Zheng et al. 2020). Gene rearrangements across the four plastomes were analysed using Mauve Alignment v2.4.0 (Darling et al. 2004) implemented in Geneious Prime v9.0.2 (Kearse et al. 2012).

The 15 plastomes, ITS, and ETS sequences have been officially submitted in GenBank (see Supplementary Table S1).

### Comparison of plastome structure

Comparative genomic analyses were conducted on 14 plastomes. The borders between inverted repeat (IR) and single-copy (SC) regions were visualized using CPJSdraw (Li et al. 2023). Sequence divergence was assessed using the mVISTA programme in Shuffle-LAGAN mode (Frazer et al. 2004), with *E. nuda* as the reference.

For codon usage analyses, 53 common SC CDS were extracted from these 14 plastomes after removing CDS <300 bp long (Wright 1990) and concatenated using PhyloSuite v1.2.2 (Zhang et al. 2020). Relative synonymous codon usage (RSCU) was calculated using the CodonW v1.4.2 programme (Peden 2000), and visualization was implemented in TBtools (Chen et al. 2020) through heatmap construction.

### Phylogenetic analyses

To reconstruct the phylogeny of *Eriocycla* and *Seseli* species, three datasets were assembled and processed: (i) nrDNA: ITS + ETS from 34 Apiaceae taxa. Fifteen ETS sequences were newly generated; the remainder was sourced from NCBI. Sequences were aligned using MAFFT v7.308 (Katoh and Standley 2013) and concatenated with PhyloSuite v1.2.2 (Zhang et al. 2020). (ii) Plastid fragments: The *matK* gene, *rbcL* gene, and *rps16* intron from 35 taxa. All sequences were obtained from NCBI. Alignment and concatenation followed the same MAFFT-PhyloSuite workflow. (iii) CDS: 79 shared SC CDS extracted from 42 complete plastomes. All plastomes were sourced from NCBI, including 15 generated by our team. CDS regions were aligned and concatenated using PhyloSuite v1.2.2 (Zhang et al. 2020; Supplementary Table S1).

Maximum likelihood (ML) analyses were conducted in RAxML v8.2.8 (Stamatakis 2014) based on the best-fit GTR + GAMMA model with 1000 bootstrap replicates. The Bayesian inference was performed using MrBayes v3.2.7 (Ronquist et al. 2012) after the program ModelTest v3.7 (Posada and Crandall 1998) calculated the best-fitting models of nucleotide substitutions under the Akaike information criterion (AIC). Four Markov chains were run for 10 million generations, sampling trees at 1000 generations intervals following a 25% burn-in. Consensus topologies from both analyses were visualized and annotated in FigTree v1.4.2 (Rambaut 2015).

## Results

### Morphological characteristics

**Figure 1.**
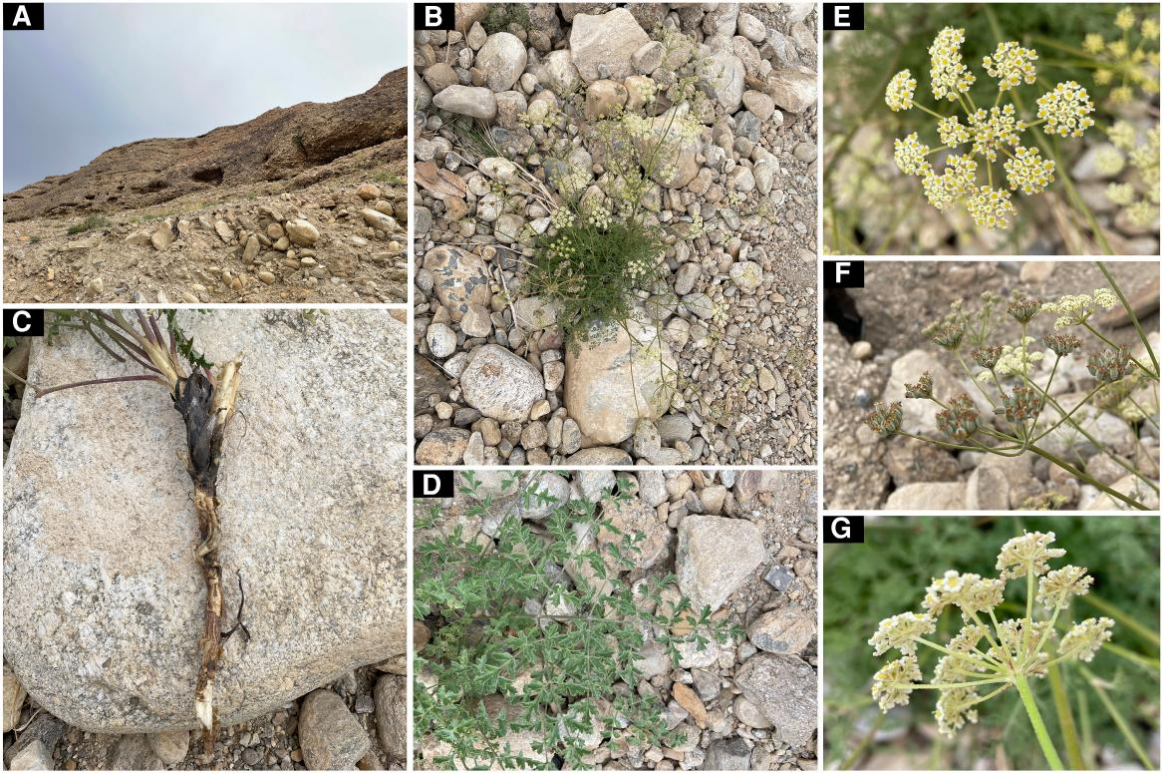
*Eriocycla nuda*. (a) Habit, (b) general morphology, (c) root, (d) basal leaves, (e) inflorescence, (f) infructescence, (g) bracts, and bracteoles.

**Figure 2.**
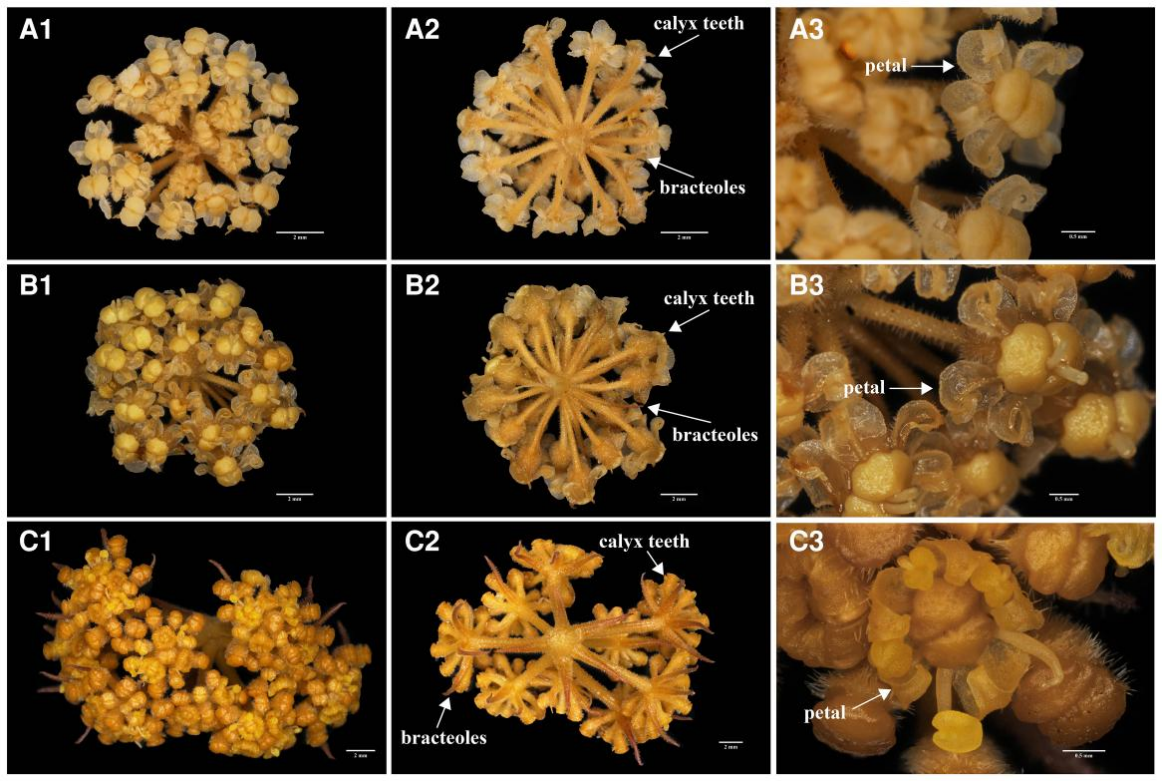
Morphology of bracteoles, petals and calyx teeth. (a) *E. nuda*, (b) *S. nortonii*, (c) *S. delavayi*.

Field investigations and inflorescence observations of *E. nuda* revealed significant morphological divergence from the previous description of *S. tortuosum* (Doğan Güner and Duman 2013). Key distinguishing traits included the absence of fibrous remnant sheaths at stem bases (Fig. 1c), contrasting with its presence in *S. tortuosum*. Additionally, *E. nuda* exhibited three to five linear bracts (Fig. 1g) compared with the absence of bracts in *S. tortuosum*, along with basally free bracteoles (Fig. 2) rather than connate. Floral morphology further differentiated the taxa, with *E. nuda* possessing pale yellow petals and prominent calyx teeth, whereas *S. tortuosum* displayed pale purple petals and obsolete calyx teeth. Notably, these traits displayed exceptional consistency among *E. nuda*, *E. pelliotii*, *S. nortonii*, and *S. delavayi*, while distinctly differentiating them from other examined *Seseli* species (see Supplementary Table S2).

Fruit morphology analyses revealed both shared and divergent characteristics. The mericarps of *E. nuda*, *S. delavayi*, and *S. nortonii* exhibited an oblong shape, while *E. pelliotii* displayed an ovoid form. Compression patterns further differentiated the taxa: *E. nuda* and *E. pelliotii* showed dorsal compression, whereas *S. delavayi* and *S. nortonii* were laterally compressed. All four species shared pubescent fruits, rounded ribs, and identical vittae configurations, with one vittae per furrow and two on commissure (see Supplementary Fig. S1).

**Figure 3.**
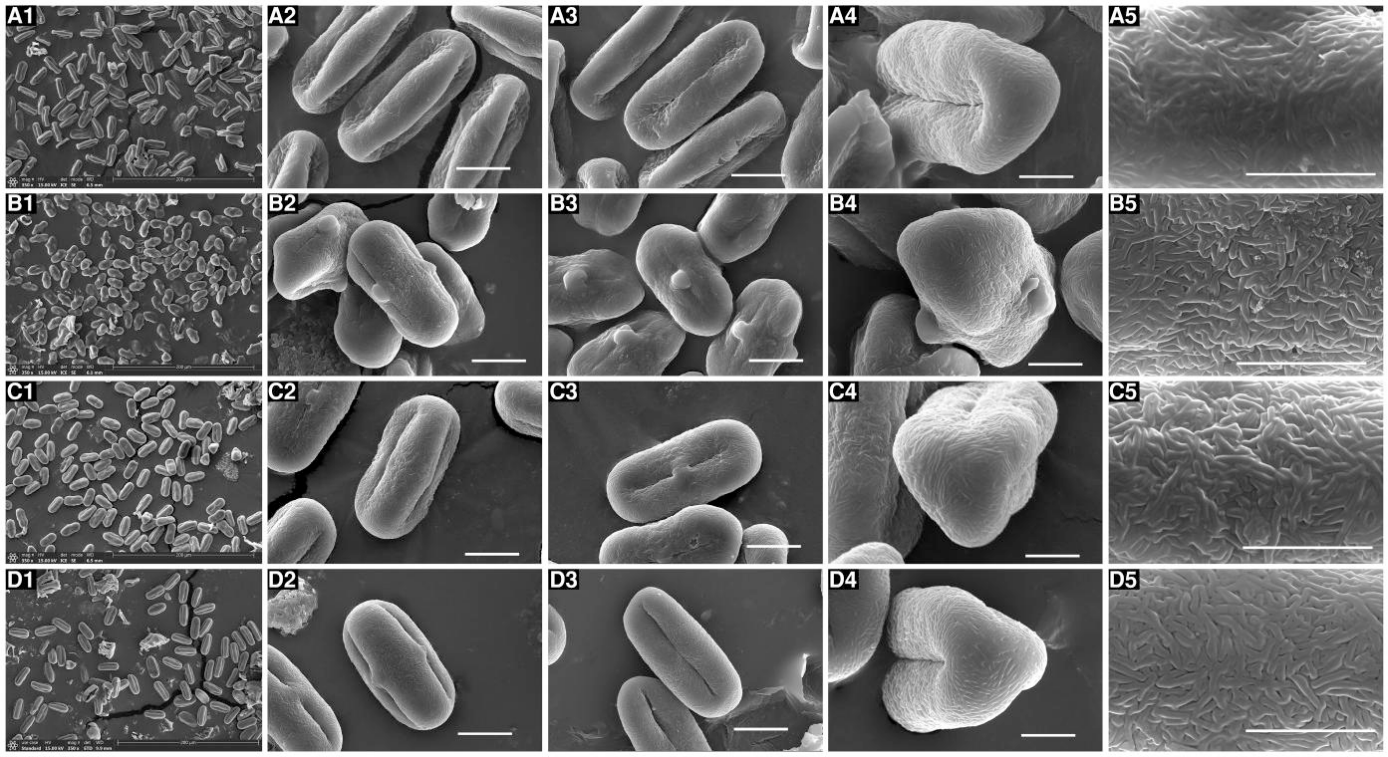
The overview, equatorial view, germ furrow, polar view and exine ornamentation of pollen grains. (a) *E. nuda*, (b) *E. pelliotii*, (c) *S. nortonii*, (d) *S. delavayi*.

The investigations of pollen morphology provided additional diagnostic evidence: while *S. tortuosum* exhibits short rod-like pseudo-cerebroid ornamentation in both equatorial and polar views (Degtjareva et al. 2011), all four studied species exhibit striato-cerebroid ornamentation in equatorial view and elongato-reticulate ornamentation in polar view (Fig. 3). However, polar perforations were observed in *E. nuda* and *S. delavayi*, contrasting with their absence in *E. pelliotii* and *S. nortonii*. In addition, the quantitative pollen metrics of these four species were divergent, with polar axis lengths of 24.70–33.63 μm, equatorial axis lengths of 12.07–17.46 μm, and *P*/*E* axis ratios of 1.64–2.05 (Table 1).

**Table 1.** Pollen features of four species.

Taxa	*E. nuda*	*E. pelliotii*	*S. nortonii*	*S. delavayi*
Equatorial view	Superrectangle	Superrectangle	Superrectangle	Subrectangular
Polar view	Trilobate circular	Subtriangular	Trilobate circular	Trilobate circular
Exine ornamentation	Striate cerebriod	Striate cerebriod	Striate cerebriod	Striate cerebriod
Polar ornamentation	Elongate reticulate, perforate	Elongate reticulate	Elongate reticulate	Elongate reticulate, perforate
Aperture	Pleurotreme	Pleurotreme	Pleurotreme	Pleurotreme
*P* (μm)	30.61 (29.10–32.57)	24.70 (23.41–25.97)	33.63 (31.47–34.94)	26.62 (25.36–28.20)
*E* (μm)	17.46 (16.84–18.83)	12.07 (11.53–13.15)	16.68 (15.78–18.10)	16.21 (15.47–17.15)
*P*/*E*	1.76	2.05	2.02	1.64

### Plastome features of *Eriocycla nuda*, *Eriocycla pelliotii*, *Seseli nortonii*, and *Seseli delavayi*

**Figure 4.**
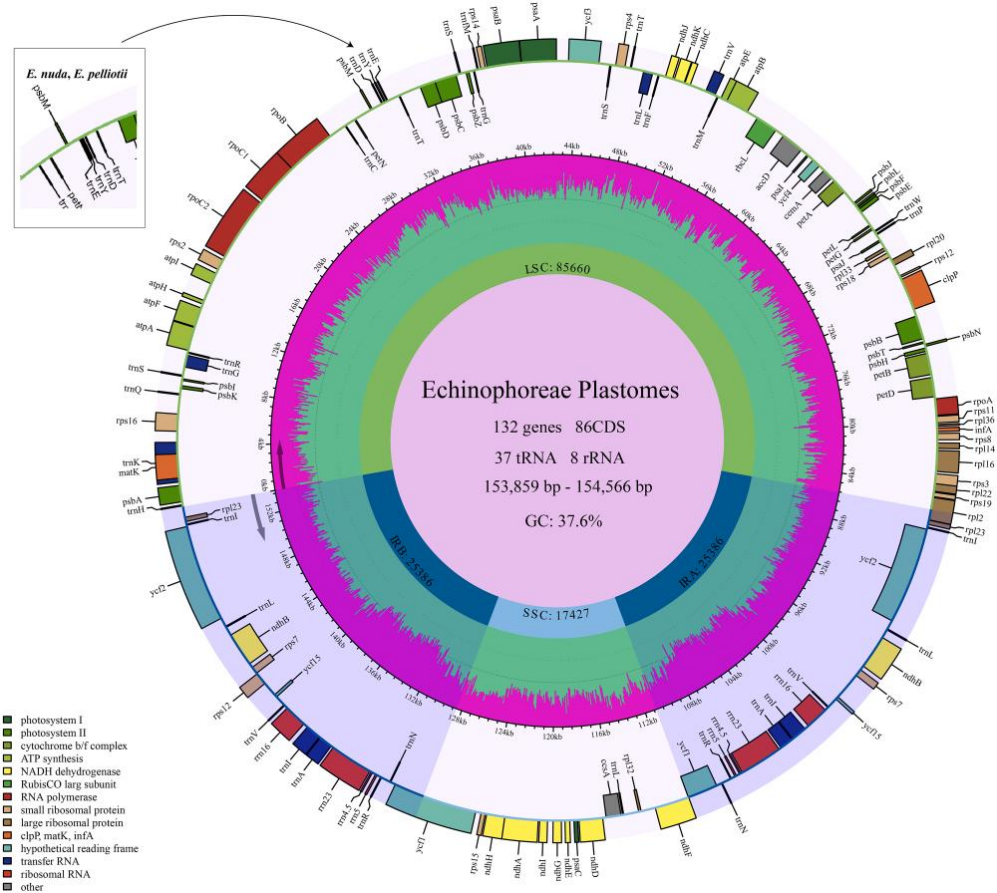
Plastome map of *E. nuda*, *E. pelliotii*, *S. nortonii*, and *S. delavayi*. Genes positioned outside the outer circle are transcribed in a clockwise direction, while those inside are transcribed counterclockwise. Functional groups of genes are distinguished by colour coding. Annotations in the upper left highlight a gene inversion event specific to the regions indicated for *E. nuda* and *E. pelliotii*.

The plastomes of these four taxa ranged in length from 153 859 bp (*S. delavayi*) to 154 566 bp (*E. pelliotii*) (Fig. 4). All plastomes exhibited the typical quadripartite structure, comprising a pair of IRs, (25 104–25 386 bp) separating the large SC (LSC, 85 660–86 920 bp) and small SC (SSC, 17 427–17 465 bp) regions (see Supplementary Table S3). The total GC content was consistent at 37.6%, with IR regions demonstrating higher GC values (42.8–42.9%) compared with LSC (35.7–35.8%) and SSC (31.1–31.2%) regions. Each plastome contained 134 genes, including 86 CDS, 37 tRNA genes, and 8 rRNA genes. Mauve alignment revealed an inversion of the *trnY–GUA*, *trnD–GUC*, and *trnE–UUC* genes in *E. nuda* and *E. pelliotii* (see Supplementary Fig. S2).

### Comparative analyses of plastomes

**Figure 5.**
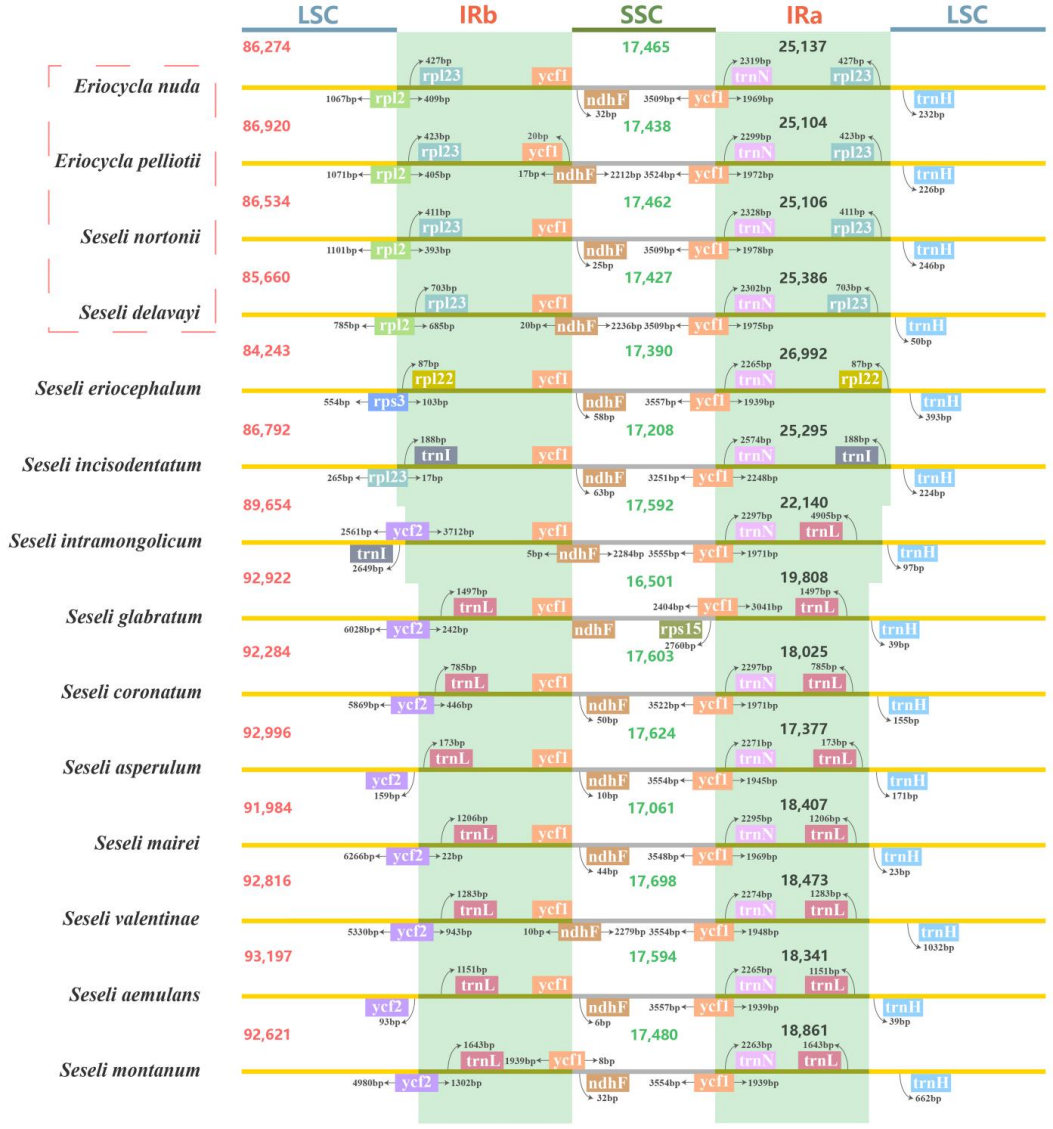
Comparison of the boundary positions of LSC, SSC, and IR regions for 14 plastomes. The coloured boxes represent functional genes and truncated fragments. The figure does not indicate the sequence length and only highlights the relative changes at or near the IR/SC boundaries.

The LSC/IRb (JLB) and IRa/LSC (JLA) borders of *E. nuda* and *E. pelliotii* were located within the *rpl12* gene and between the *rpl23* and *trnH–GUG* genes, respectively, a pattern distinct from the other ten *Seseli* species but highly congruent with *S. nortonii* and *S. delavayi* (Fig. 5). Notably, *S. delavayi* exhibited the shortest *rpl12* gene segment in the LSC region (785 bp) and the longest in the IRb region (685 bp) among the analysed plastomes. Two distinct types of IRb/SSC (JSB) borders were identified in these 14 plastomes: the first type occurred within the *ndhF* gene in *E. pelliotii*, *S. delavayi*, *S. valentinae*, and *S. intramongolicum*, while the second type was positioned between the *ycf1* and *ndhF* genes in *E. nuda*, *S. nortonii*, *S. eriocephalum*, *S. glabratum*, *S. coronatum*, *S. asperulum*, *S. mairei*, *S. aemulans*, *S. intramongolicum*, and *S. incisodentatum*. The JLA borders of *S. nortonii*, *S. delavayi*, *E. nuda*, and *E. pelliotii* were located between the *rpl23* and *trnH–GUG* genes, whereas this junction occurred between *rpl22* and *trnH–GUG* genes in *S. eriocephalum* and between the *trnL–CAA* and *trnH–GUG* genes in other *Seseli* species.

The mVISTA results demonstrated higher sequence conservation in coding regions compared with non-coding regions, with inverted IR regions being more conserved than SC regions (see Supplementary Fig. S3). When using *E. nuda* as the reference, *S. nortonii* and *S. delavayi* exhibited substantial sequence divergence from other *Seseli* species, showing closer affinity to *Eriocycla* taxa. Polymorphisms were observed in highly divergent regions, including *rps16–trnQ–UUG*, *trnE–UUC–trnT–GGU*, *trnT–UGU–trnL–UAA*, *rpl32–trnL–UAG*, and *petA–psbJ*.

Analysis of 53 selected CDS across the 14 species identified 21 168–21 212 codons, with leucine (Leu) displaying the highest number of codons (2221–2244) and cysteine (Cys) showing the lowest (215–224) (see Supplementary Fig. S4). The RSCU values ranged from 0.33 to 2.02, where the codons AUG and UGG exhibited RSCU values of 1.00, indicating no codon usage bias. Among the 30 codons with RSCU > 1.00, all terminated with a purine (A/U) except UUG. The arginine codon CGA showed RSCU values of 1.35–1.36 in *E. nuda*, *E. pelliotii*, *S. nortonii*, and *S. delavayi*, compared with values ≥1.38 in the remaining 10 *Seseli* species.

### Phylogenetic analyses

**Figure 6.**
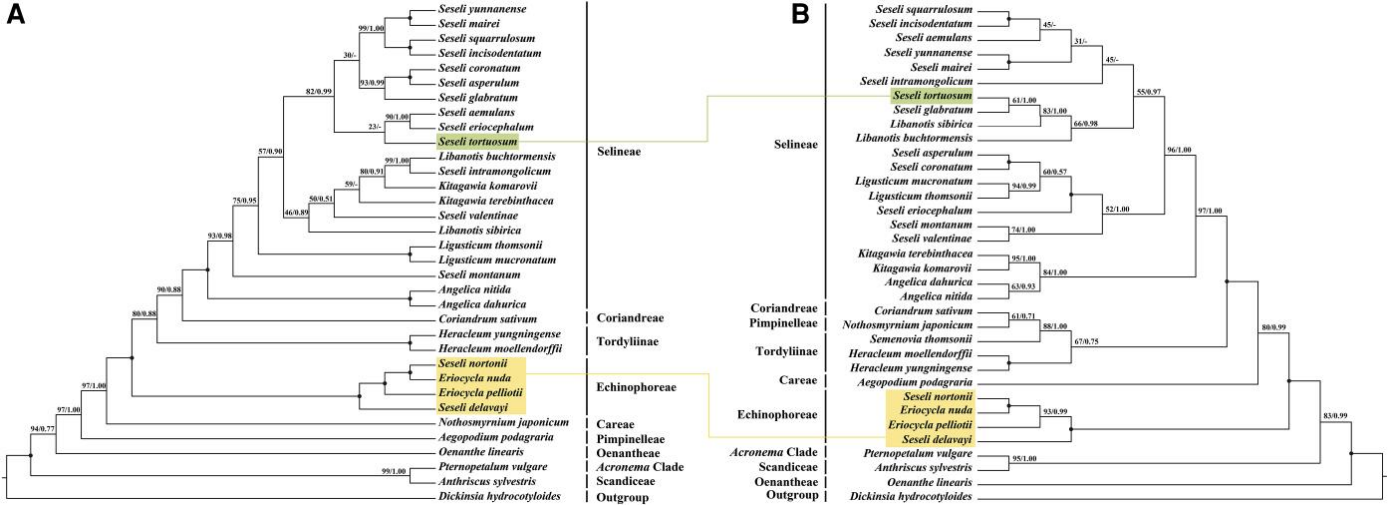
Phylogenetic trees inferred from Bayesian inference and Maximum Likelihood. Branch support was assessed using bootstrap percentage of ML and the followed posterior probability of BI. The solid circle represents maximum support in both two analyses (Bootstrap value = 100%, Posterior probability = 1.00); (−) represents the values <50%. (a) Phylogenetic tree of 34 Apioideae taxa based on nrDNA, (b) phylogenetic tree of 35 taxa based on plastome fragments.

**Figure 7.**
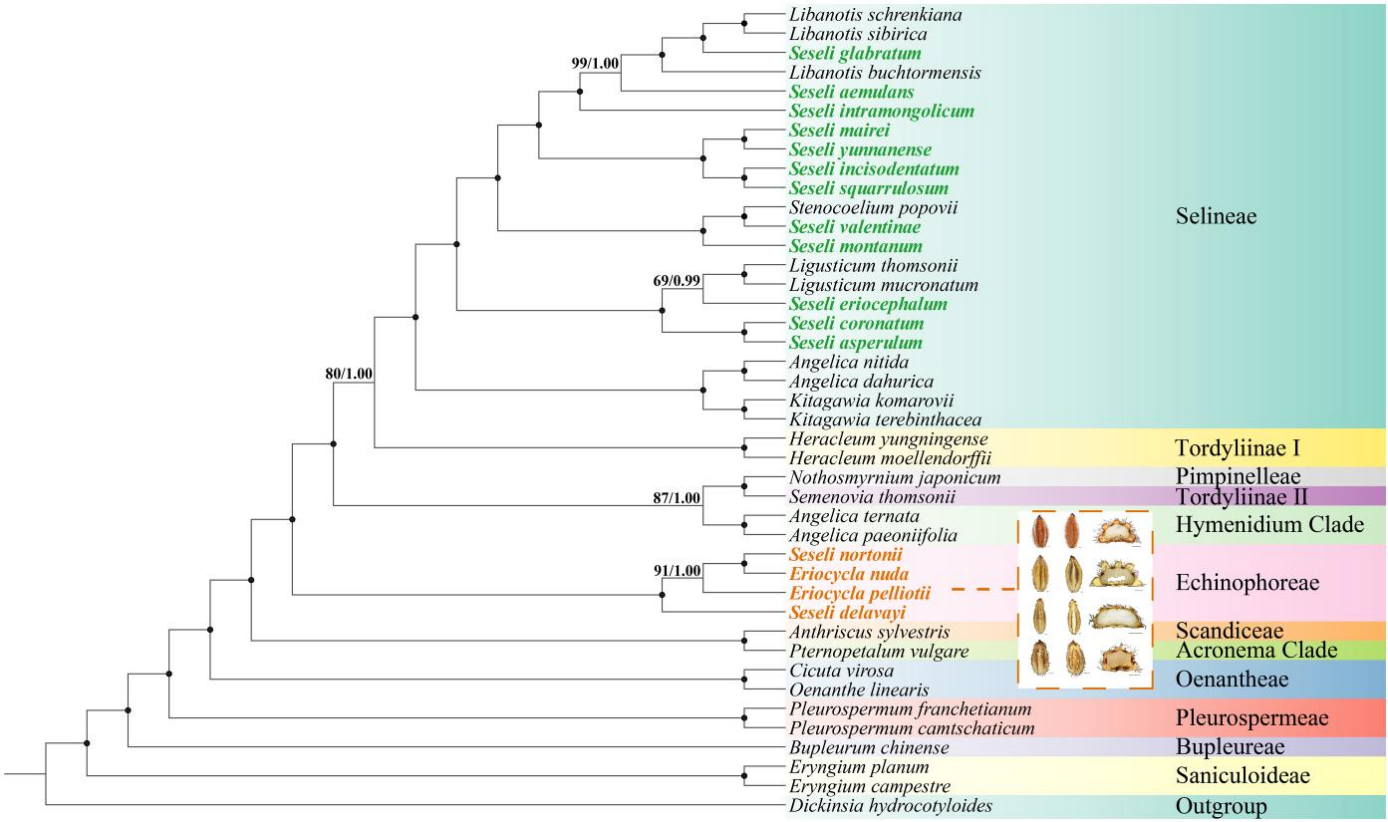
Phylogenetic tree inferred from 42 taxa based on 79 shared CDS, with carpological characteristics annotated at corresponding nodes. Scale bars: 0.5 mm (dorsal side views), 0.5 mm (transverse sections).

Phylogenetic reconstructions based on nrDNA and plastid fragments datasets robustly supported the monophyly of *E. nuda*, *E. pelliotii*, *S. nortonii*, and *S. delavayi* (nrDNA: BS = 100%, PP = 1.00, Fig. 6a; plastid fragments: BS = 100%, PP = 1.00, Fig. 6b), clustering these taxa within the tribe Echinophoreae. In contrast, the type species *S. tortuosum* and other *Seseli* members resolved within the tribe Selineae, forming a highly divergent paraphyletic group, demonstrating distant phylogenetic affinities (nrDNA: BS = 100%, PP = 1.00; plastid fragments: BS = 97%, PP = 1.00). Furthermore, the high-resolution CDS tree generated through concatenation methods exhibited identical topologies (BS = 100%, PP = 1.00, Fig. 7). Notably, across all three phylogenetic trees, *S. nortonii* formed a well-supported sister group with *E. nuda* (nrDNA: BS = 100%, PP = 1.00; plastid fragments: BS = 100%, PP = 1.00; CDS: BS = 100%, PP = 1.00) (Figs 6 and 7), the type species of *Eriocycla*.

## Discussion

In this study, we reconstructed the phylogenetic relationships of *Eriocycla* and *Seseli* based on 3 datasets (34 nrDNA sequences, 35 plastid fragments, and 42 plastomes) and obtained high-resolution trees for these 2 genera. *Eriocycla nuda* and *E. pelliotii*, two species previously classified within *Seseli* sect. *Eriocycla*, formed a well-supported monophyletic clade within the tribe Echinophoreae. This finding contradicts the treatment proposed by Pimenov and Kljuykov (2000). Based on fruit anatomical analyses of *E. nuda* and *E. pelliotii*, they concluded that traits such as fruit compression type, pubescence, and the number of vittae align these species most closely with *Seseli*, leading them to establish the *Seseli* sect. *Eriocycla*. However, the Apiaceae family is notoriously one of the most complicated families of flowering plants, with highly diverse fruit characteristics (Pimenov et al. 2019, Wojewódzka et al. 2019, Kljuykov et al. 2021), particularly within world-wide complex genera like *Seseli*, and we cannot exclude the influence of homoplasy and hybridization. Our results of the phylogeny also indicate we could not simply merge *Eriocycla* into *Seseli*. Furthermore, based on combined morphological and molecular evidence, Lyskov et al. (2020, 2022) transferred one species from *Seseli* sect. *Eriocycla* to *Semenovia* Regel & Herder and described two others as the new genus *Shomalia* Lyskov. The frequent taxonomic revisions, combined with the profound phylogenetic distance separating its core species (*E. nuda*, *E. pelliotii*) from *Seseli*, strongly indicate that the concept of *Seseli* sect. *Eriocycla* is no longer tenable. Our phylogenetic analyses also revealed that two *Seseli* taxa, *S. nortonii* and *S. delavayi*, resolve within the tribe Echinophoreae, while other *Seseli* species studied, including the type species *S. tortuosum*, fall into the tribe Selineae. The phylogeny of these taxa splits definitively into two major clades: one within Echinophoreae comprising *E. nuda*, *E. pelliotii*, *S. nortonii*, and *S. delavayi*; and another scattered within Selineae encompassing all remaining *Seseli* species examined. Comparative plastome analyses largely corroborated this division, the four Echinophoreae species share congruent plastome structures at the LSC/IRb (JLB: within the *rpl12* gene) and IRa/LSC (JLA: between *rpl23* gene and *trnH–GUG* genes) borders, a type distinct from other *Seseli* members (JLB: within *ycf2* gene; JLA: between *trnL–CAA* and *trnH–GUG* genes). Regarding morphology, *E. nuda*, *E. pelliotii*, *S. delavayi*, and *S. nortonii* share key morphological characteristics absent in the other *Seseli* species studied, including glabrous stem bases, basally free bracteoles, and prominent calyx teeth (Supplementary Table S2). According to Doğan Güner et al. (2011) on the pollen features of *Seseli*, *S. tortuosum* exhibits short rod-like pseudo-cerebroid ornamentation in both equatorial and polar views, whereas in this study, the four Echinophoreae taxa displayed striato-cerebroid ornamentation in equatorial view and elongato-reticulate ornamentation in polar view.

Pimenov and Kljuykov (2000) treated *S. nortonii* as a synonym of *E. nuda* due to similarities in carpological features. These two species formed a solid sister group in our nrDNA-based and cpDNA-based trees, but a unique inversion of the *trnY–GUA*, *trnD–GUC*, and *trnE–UUC* genes was detected in *E. nuda* but absent in *S. nortonii*. Significant morphological divergences also exist between the two taxa: *S. nortonii* possesses densely hispid and thickly leathery basal leaves with laterally compressed fruits, contrasting with the pubescent and thickly membranous leaves and dorsally compressed fruits of *E. nuda*. These results demonstrate that adopting *S. nortonii* as a synonym of *E. nuda* is unsuitable.

Therefore, based on integrated evidence from phylogenetics, plastome structures, and morphological characteristics, we formally propose reinstating *Eriocycla* as an independent genus. *S. nortonii* and *S. delavayi* are hereby transferred to *Eriocycla* as *Eriocycla nortonii* comb. nov. and *Eriocycla delavayi* comb. nov., respectively. These findings provide new insights into the phylogeny of the tribe Echinophoreae. Regarding *Seseli*, one of the largest polyphyletic groups in the Apiaceae family, Cai et al. (2022) proposed defining this genus in a narrow sense (*Seseli* s.s.), comprising nine core members. Nevertheless, critical gaps in morphological and molecular data, particularly for the type species *S. tortuosum*, mean achieving a scientifically robust reclassification of *Seseli* in its entirety remains an arduous task requiring expanded sampling and multidisciplinary analyses.

## Conclusion

This study reconstructs the phylogeny of *Seseli* and *Eriocycla* through three datasets: (i) 34 nrDNA(ITS + ETS); (ii) 35 plastid fragments (*matK* + *rbcL* + *rps16* intron); (iii) 42 plastomes (79 shared CDS). These analyses robustly resolve *E. nuda*, *E. pelliotii*, *S. nortonii*, and *S. delavayi* as a well-supported monophyletic group within the tribe Echinophoreae, demonstrating distant phylogenetic relationships from *S. tortuosum* and other studied *Seseli* members of the tribe Selineae. Comparative plastome analyses congruently corroborated these distinctions: the four taxa exhibited structural conservation at JLB (within the *rpl12* gene) and JLA (between the *rpl23* and *trnH–GUG* genes) borders, alongside convergent codon usage bias for the CGA codon. The genus *Eriocycla* is characterized by glabrous stem bases, basally free bracteoles, and prominent calyx teeth, which clearly differentiate it from *S. tortuosum* and other *Seseli* species. Integrating morphological and molecular evidence confirms *Seseli* sect. *Eriocycla* does not constitute a natural group. Thus, we propose reinstating *Eriocycla* as an independent genus and transferring *S. nortonii* and *S. delavayi* to this genus, recognizing them as *E. nortonii* comb. nov. and *E. delavayi* comb. nov. In summary, our study resolved a long-standing controversy in Apiaceae family, providing new insights into the phylogenetic relationships of the tribe Echinophoreae and establishing a reference framework for future revisions of *Seseli*.

## Supplementary Material

plaf045_Supplementary_Data

## Figures and Tables

**Figure 8.**
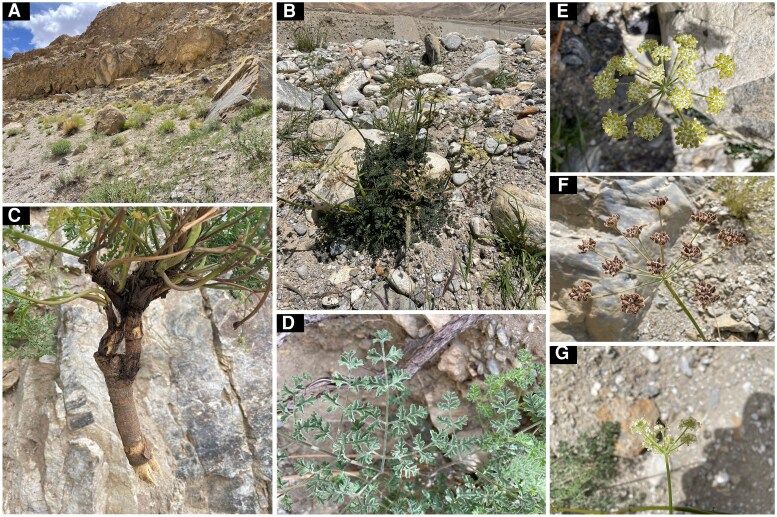
*Seseli nortonii*. (a) Habit, (b) general morphology, (c) root, (d) basal leaves, (e) inflorescence, (f) infructescence, (g) bracts and bracteoles.

**Figure 9.**
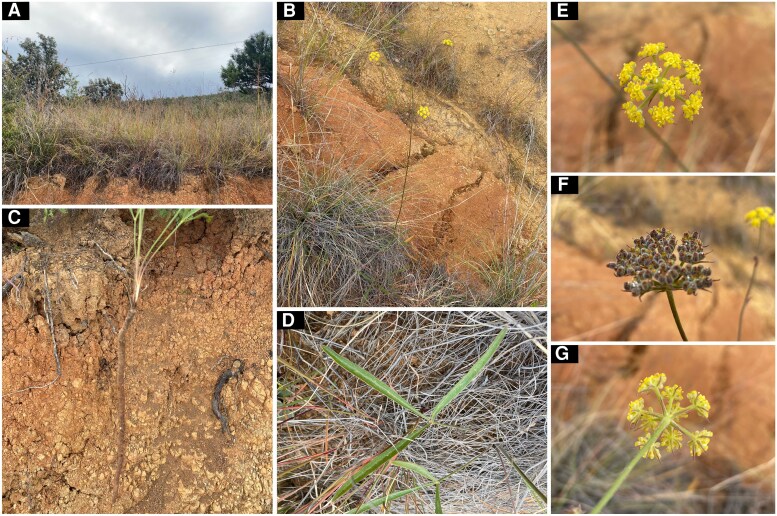
*Seseli delavayi*. (a) Habit, (b) general morphology, (c) root, (d) basal leaves, (e) inflorescence, (f) infructescence, (g) bracts and bracteoles.

## Data Availability

All newly generated DNA sequences have been submitted to NCBI (https://www.ncbi.nlm.nih.gov), and the GenBank accession numbers with other detailed information are provided in Supplementary Table S1.

## Appendix

### Taxonomic treatment

***Eriocycla* Lindl.**

**Type species:**  *Eriocycla nuda* Lindl. (Fig. 1)

***Diagnosis***

This genus can be easily distinguished from *Seseli* by glabrous stem bases (vs. fibrous remnant), numerous bracts (vs. absent or 1–2), basally free bracteoles (vs. connate), prominent calyx teeth (vs. obsolete), and rounded mericarp ribs (vs. keeled or filiform).

***Eriocycla nortonii* (Fedde ex H.Wolff) J.Cai & S.D.Zhou, comb. nov.** (Fig. 8)

**Basionym:**  *Seseli nortonii* Fedde ex H.Wolff (1930, p. 329)

**Type:** China, Xizang: Dingri County, Kada Valley, crevice, 3600–4000 m a.s.l., 16 June 1922, Norton 108 (holotype: K!—barcode K000697458).

***Diagnosis***

*Eriocycla nortonii* closely resembles *E. nuda* but differs by the following characters: basal leaves densely covered with white-hispid hairs (vs. white pubescence), ultimate segments thickly coriaceous (vs. membranous to papery), involucral bracts 10 (vs. 3–5), fruits laterally compressed (vs. dorsally compressed), and pollen lacking polar perforations (vs. present).

***Description***

Plants 30–70 cm. Roots cylindrical, yellowish-brown. Stems solitary or clustered, purplish at base, hollow, striate, sparsely pubescent, branched from lower parts; stem base lacking fibrous remnants. Basal leaves numerous; petioles long, sheaths membranous-margined, hirsute; leaf blade broadly rhombic, 2–3-pinnate; ultimate segments ovate, margins serrate; rachis and abaxial surface densely white-hispid, adaxial surface sometimes glabrous; texture thickly coriaceous. Compound umbels with elongated peduncles; involucral bracts 10, linear-lanceolate, densely white-hispid, shorter than rays; rays 10–15, unequal; umbellules many-flowered; bracteoles 10, linear-lanceolate, densely hirsute, basally free. Petals pale yellow, suborbicular, apex emarginate with inflexed lobule, abaxially densely white-hispid. Calyx teeth subulate, prominent. Styles short, erect; stylopodium depressed-conical. Mericarps oblong, densely white-hispid, laterally compressed; ribs rounded; vittae one per furrow, two on commissure.

***Distribution and habitat***

Endemic to Dingri County, Xizang, China. Grows in arid rocky slopes or crevices at 3600–4000 m a.s.l.

***Additional specimens examined***

China, Xizang: Dingri County, Qudang Village, 28° 07′ N, 87° 31′ E, 3737 m a.s.l., 4 August 2022, J. Cai & J.Q. Lei CJ202208040101 (SZ); Qudang Village, 28° 25′ N, 87° 38′ E, 3772 m a.s.l., 4 August 2022, J. Cai & J.Q. Lei CJ202208040102 (S.-D.Z.); Zhaxizong Village, 28°34′N, 87°19′E, 3947 m a.s.l., 4 August 2022, J. Cai & J.Q. Lei CJ202208040201 (S.-D.Z.).

***Eriocycla delavayi* (Franch.) J. Cai & S. D. Zhou, comb. nov.** (Fig. 9)

**Basionym:** Seseli delavayi Franch. (1894: 130).

**Type:** China, Yunnan: Heqing County, Lo-ho-chan Mountain, calcareous hills above Che-tong near Tapintze, 1500 m a.s.l., 4 September 1885, J.M. Delavay 2024 (lectotype: P!—barcode P04197843; isotype: K!—barcode K000697465)

***Diagnosis***

*Eriocycla delavayi* is distinguished from other *Eriocycla* species by its ternatisect basal leaves with lanceolate segments, subequal rays, ovoid fruits densely covered with white-hispid hairs, and elliptic to subrectangular pollen.

***Description***

Plants 50–90 cm, densely white-hispid throughout. Roots conical, yellowish-brown, woody. Stems solitary, branched above middle, cylindrical, densely white-hispid; stem base lacking fibrous remnants. Basal leaves ternatisect, segments sessile, linear-lanceolate, parallel-veined, both surfaces densely white-hispid. Compound umbels with elongated peduncles; involucral bracts 5–10, linear-lanceolate, acuminate, densely white-hispid, shorter than rays; rays 8–10, subequal; bracteoles 5–7, linear-lanceolate, densely hispid, basally free, longer than pedicels. Petals yellow, obovate, apex emarginate with inflexed lobule, abaxially densely white-hispid. Calyx teeth subulate, prominent. Styles very short, concealed within stylopodium; stylopodium conical. Mericarps oblong, densely white-hispid, laterally compressed; ribs rounded, obscured by indumentum; vittae one per furrow, two on commissure.

***Distribution and habitat***

Endemic to Yunnan, China (Heqing, Yuanmou). Grows on sunny grassy slopes at 1200–1800 m a.s.l.

***Additional specimens examined***

China, Yunnan: Heqing County, Tapintze, 1500 m a.s.l., 4 September 1885, J.M. Delavay 2024 (P); Tapintze, 1700 m a.s.l., 16 September 1929, R.C. Qin 24646 (PE); Tapintze, 1400 m a.s.l., 12 August 1963, 6463 (KUN); Yuanmou County, Wuguo Village, 1701 m a.s.l., 16 November 2021, J. Cai & H.H. Qin CJ0202111160201 (S.-D.Z.).

**Key to species of *Eriocycla***

1a. Basal leaves ternatisect, ultimate leaf segments linear-lanceolate; mericarp laterally compressed, densely hispid*E. delavayi*

1b. Basal leaves (1-)2-pinnate, ultimate leaf segments ovate; mericarp laterally or dorsally compressed, densely pubescent2

2a. Mericarp oblong, laterally compressed*E. nortonii*

2b. Mericarp oblong or ovoid, dorsally compressed3

3a. Mericarp oblong; ultimate leaf segments deeply serrate on margins*E. nuda*

3b. Mericarp ovoid; ultimate leaf segments sparsely serrate on margins*E. pelliotii*
