# Supplementary material for: Taxonomic position of Eriocycla (Apiaceae): insights from molecular and morphological evidence
Source: AoB Plants. 2025 Aug 28;17(5):plaf045. doi: 10.1093/aobpla/plaf045 (PMC12459255; doi:10.1093/aobpla/plaf045)
Supplement: plaf045_Supplementary_Data [file plaf045_supplementary_data.zip › Supporting information.pdf]

Supporting Information

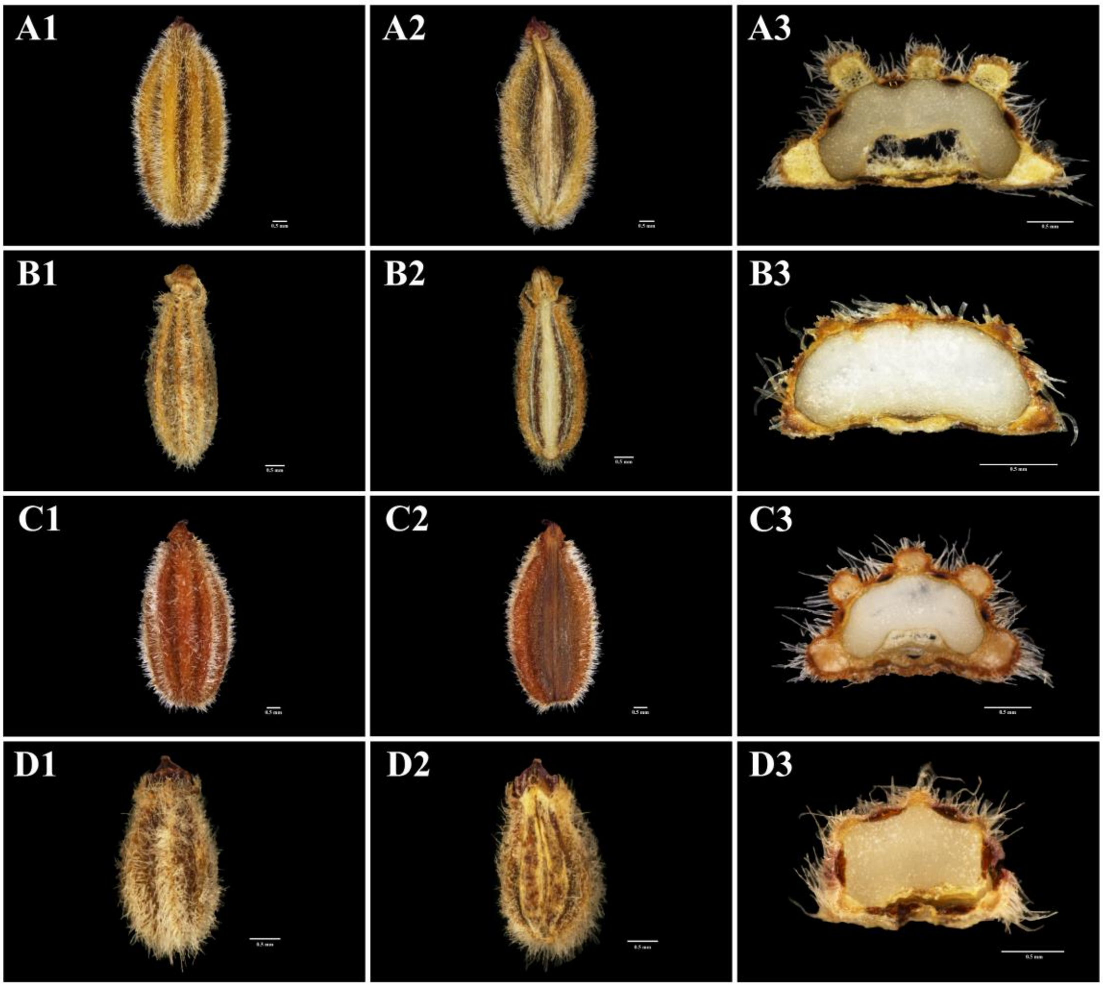

Figure S1. The dorsal view, commissural side view and transverse section view of mericarps. (A) *E. nuda*, (B) *E. pelliotii*, (C) *S. nortonii*, (D) *S. delavayi*.

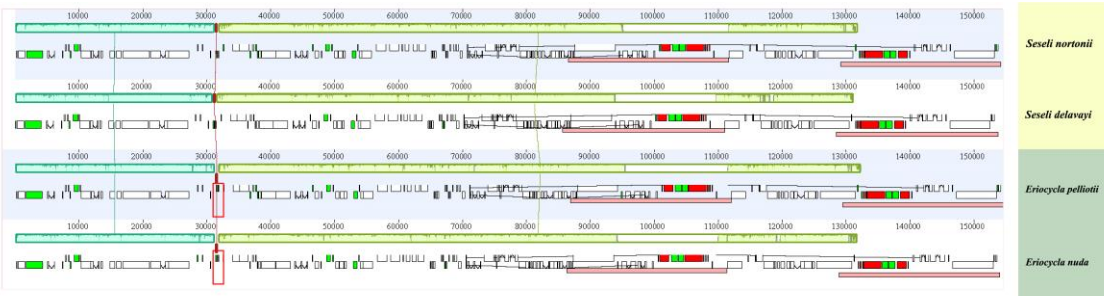

Figure S2. Mauve alignment of four plastomes. Local collinear blocks within each alignment are represented by blocks of the same color connected with lines.

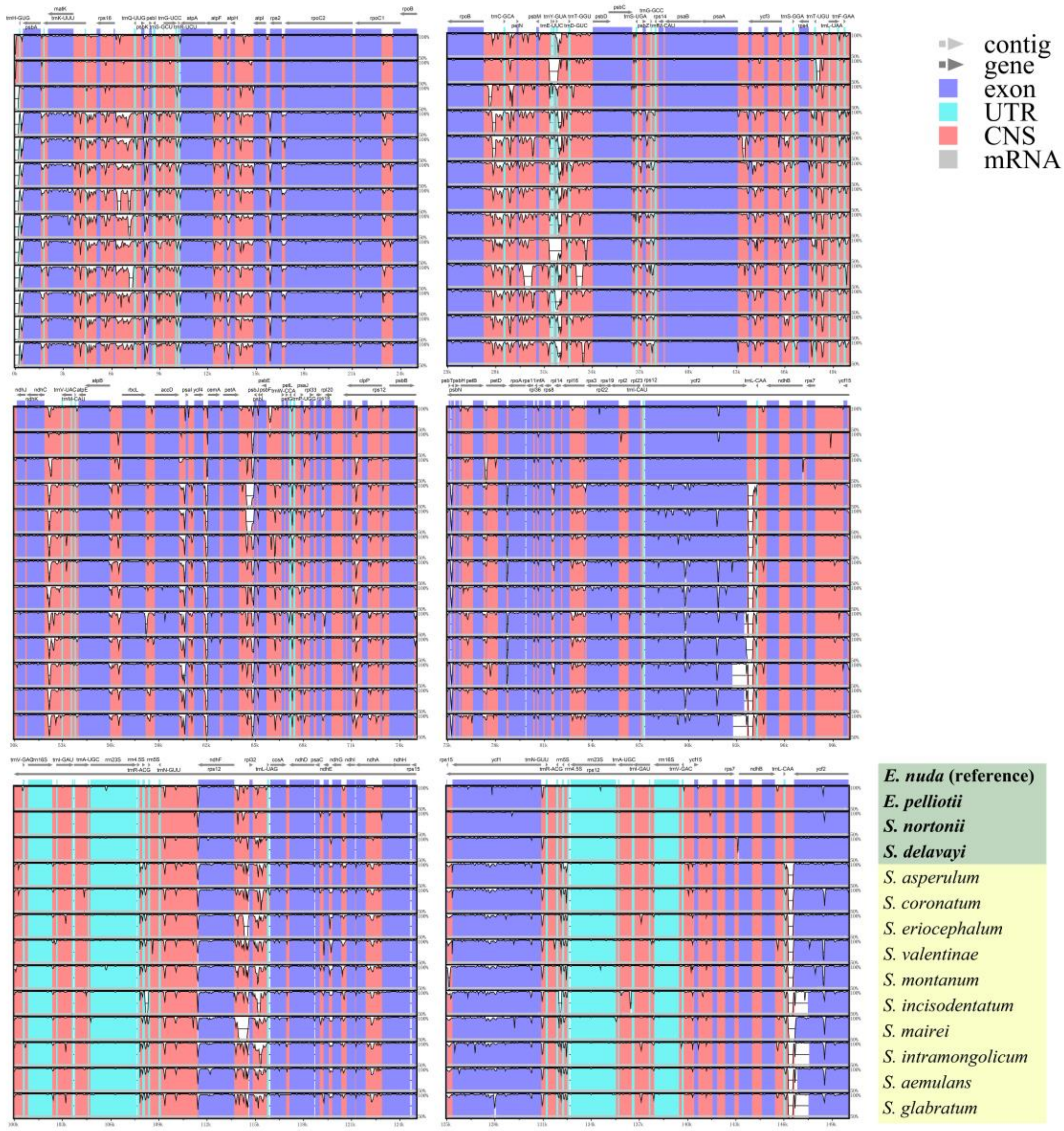

Figure S3. The sequence identity plots of 14 plastomes generated by mVISTA. *E. nuda* as the reference.

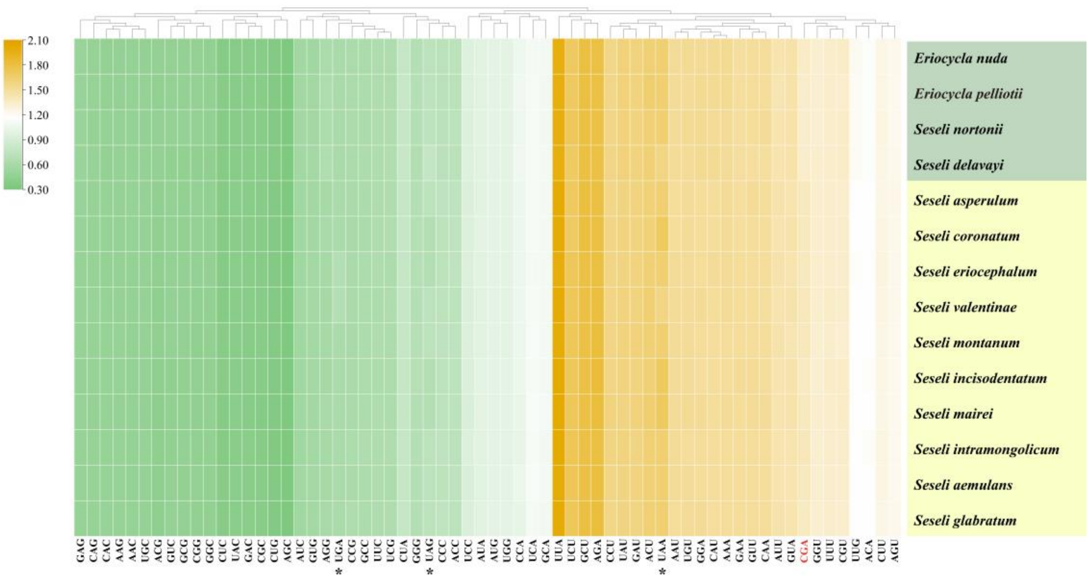

Figure S4. The RSCU values of 53 CDS for 14 species. The terminator codons were marked with (*).
